# Supplementary material for: Prescription of anti-influenza drugs in Japan, 2014–2020: A retrospective study using open data from the national claims database
Source: PLoS One. 2023 Oct 4;18(10):e0291673. doi: 10.1371/journal.pone.0291673 (PMC10550188; doi:10.1371/journal.pone.0291673)
Supplement: Attachment — Submitted filename: Review Comments.pdf [file pone.0291673.s001.pdf]

Review Comments to the Author

Influenza is an important infectious disease, and this study is considered to be a useful one. However, in the current manuscript, the methods presented by the authors did not seem to support the story of their study.

Particularly, I feel that the most important point of this study, the method of estimating the number of patients based on the amount of anti-influenza drug prescriptions, is based on the authors' own speculation, and the authors have not sufficiently cited previous studies or conducted sensitivity analysis to verify the validity of their own method.

On the other hand, the authors claim that the figures in this study are consistent with other studies in Japan. I also believe that there is a possibility of publication by giving full consideration to the significance, methods, and discussion of the study.

(BACKGROUND SECTION)

1. There are few citations in the literature regarding the number of influenza cases in Japan and other countries. Does this mean that there are few prior studies on influenza incidence in Japan? Appropriate prior studies need to be cited.
2. The clinical and international significance of this study is not clear. My question is: "What is the significance of counting patients?" Please specify what the academic significance of estimating the number of influenza patients and medical costs is. In the background section, you should describe how little is known from previous studies and what is newly proven in this study.
3. (line 15 of p5) Related to #2, the justification for "there is a paucity of nationwide epidemiological and economic data" is not stated. Does this mean that The National Institute of Infectious Diseases reports data are not nation-wide? The reason for paucity is an important part of the significance of this study, so please describe it in detail.

(METHODS SECTION)

4. (line 11 of p6) Is "FY" an abbreviation that appears without explanation? Also, please describe the specific time period of FY. In a flu study, it is considered of most importance what month the statistics are from, and without that information, it is difficult to conduct proper peer review. (This relates to statistical adequacy.)

5. (line 15 of p6) I could not find a relation between this sentence and this study. Most of the papers cited have nothing to do with infectious diseases.
6. (after line 2 of p7) The formula for converting the prescription volume of anti-influenza drugs to the number of patients is perhaps the most important point of this study. In particular, the number of pediatric patients cannot be ignored for influenza. Regarding children, whose weight gain is marked and individual differences are large, I could not determine whether your method of estimating the number of patients administered using national weight averages in 5-year increments was correct. It is essential to properly cite the demographic or pharmacoepidemiology literature to support this approach. I believe that a sensitivity analysis should be performed to examine the authors' assertions from multiple perspectives by varying the estimating equations. (This relates to statistical adequacy.)
7. (lines 14-20 of p7) Related to #6, the description of the validity of the authors' conversion formula prescription volume=>patient volume is lacking. The authors need to validate the conversion equation with some literature from pharmacoepidemiology or other sources.

(RESULTS SECTION)

8. The ranking of anti-influenza drug prescriptions is considered to be correct content. However, I read that it is what has been initially published in NDB Open Data and not the authors' original estimation, is this understanding correct? (Sorry if I am wrong.)

(DISCUSSION SECTION)

9. (p13) It is essential to prove the validity of the results of this study by examining in concrete detail the similarities with the results of the national survey conducted by NESID. (One of the most important points of this research.)
10. (lines 11-21 on p. 14) I understand that this description is relevant to the authors claim. However, how about stating the most important point above (proving the validity of the results of this study) in more detail?
11. (lines 1-8 on p15) How about a brief description? I feel that the clinical claims are strong compared to a weaker explanation of the validity of the results of this study.

That is all my review opinion.
