# Supplementary material for: Prescription of anti-influenza drugs in Japan, 2014–2020: A retrospective study using open data from the national claims database
Source: PLoS One. 2023 Oct 4;18(10):e0291673. doi: 10.1371/journal.pone.0291673 (PMC10550188; doi:10.1371/journal.pone.0291673)
Supplement: Attachment — Submitted filename: PONE-D-23-02107_R2_reviewer.docx [file pone.0291673.s003.docx]

I appreciate great efforts of the authors in addressing reviewers’ comments. Given the limitation of the data, I believe the findings of the study may significantly contribute to current literature and invite further investigation of anti-influenza medication prescriptions in Japan. After incorporating reviewers’ comments, I think the quality of content in the manuscript improved. However, there are some remaining points that the authors can take into consideration.

1. The Methods section seems to be lengthy and contains too much text. In describing the anti-influenza drugs, the authors can create a table for all available anti-influenza medications, with marketed years, indications, dosing, dosage forms, and their pediatric use. In addition, the authors should describe the methods to identify these drugs in NDB Open Data (i.e., by search string or NDC/ATC code, etc) and the validity of exposure ascertainment. Since the authors mentioned the presence of missing data in NDB, this can also pose a threat to the estimates.

2. The authors organized the Methods section by describing the components of the NDB Open Data rather than focusing on the components of an observational study. Please refer to the STROBE guidelines to describe the components of the study, in terms of study design, data sources, exposure, outcome/measure, covariates, statistical analysis, and sensitivity analysis. This can make the manuscript more attractive to the readers.

3. From statistical perspective, any estimation should have uncertainty. However, I could not find any uncertainty of estimates (i.e., 95% confidence intervals, standard errors). Could the authors clarify more on the estimate of annual number of prescriptions/tests or cost in the analysis?
